# Evaluate the in vitro effect of anthracycline and alkylating cytophosphane chemotherapeutics on dopaminergic neurons

**DOI:** 10.1002/cnr2.2074

**Published:** 2024-04-16

**Authors:** Darshini Desai, Mohammed Majrashi, Suhrud Pathak, Mohammed Almaghrabi, Keyi Liu, Satyanarayana R. Pondugula, Amit K. Tiwari, R. Jayachandra Babu, Jack Deruiter, Muralikrishnan Dhanasekaran

**Affiliations:** ^1^ Department of Drug Discovery and Development Harrison College of Pharmacy, Auburn University Auburn Alabama USA; ^2^ Department of Pharmacology Faculty of Medicine, University of Jeddah Jeddah Saudi Arabia; ^3^ Department of Medicinal Chemistry Faculty of Pharmacy, Taibah University Al‐Medina Saudi Arabia; ^4^ Department of Anatomy Physiology and Pharmacology, College of Veterinary Medicine, Auburn University Auburn Alabama USA; ^5^ Department of Pharmaceutical Sciences College of Pharmacy, University of Arkansas for Medical Sciences Little Rock Arkansas USA

**Keywords:** apoptosis, cyclophosphamide, dopaminergic neurotoxicity, doxorubicin, mitochondrial function, oxidative stress

## Abstract

**Background:**

Iatrogenesis is an inevitable global threat to healthcare that drastically increases morbidity and mortality. Cancer is a fatal pathological condition that affects people of different ages, sexes, and races around the world. In addition to the detrimental cancer pathology, one of the most common contraindications and challenges observed in cancer patients is severe adverse drug effects and hypersensitivity reactions induced by chemotherapy. Chemotherapy‐induced cognitive neurotoxicity is clinically referred to as Chemotherapy‐induced cognitive impairment (CICI), chemobrain, or chemofog. In addition to CICI, chemotherapy also causes neuropsychiatric issues, mental disorders, hyperarousal states, and movement disorders. A synergistic chemotherapy regimen of Doxorubicin (Anthracycline‐DOX) and Cyclophosphamide (Alkylating Cytophosphane‐CPS) is indicated for the management of various cancers (breast cancer, lymphoma, and leukemia). Nevertheless, there are limited research studies on Doxorubicin and Cyclophosphamide's pharmacodynamic and toxicological effects on dopaminergic neuronal function.

**Aim:**

This study evaluated the dopaminergic neurotoxic effects of Doxorubicin and Cyclophosphamide.

**Methods and Results:**

Doxorubicin and Cyclophosphamide were incubated with dopaminergic (N27) neurons. Neuronal viability was assessed using an MTT assay. The effect of Doxorubicin and Cyclophosphamide on various prooxidants, antioxidants, mitochondrial Complex‐I & IV activities, and BAX expression were evaluated by Spectroscopic, Fluorometric, and RT‐PCR methods, respectively. Prism‐V software (La Jolla, CA, USA) was used for statistical analysis. Chemotherapeutics dose‐dependently inhibited the proliferation of the dopaminergic neurons. The dopaminergic neurotoxic mechanism of Doxorubicin and Cyclophosphamide was attributed to a significant increase in prooxidants, a decrease in antioxidants, and augmented apoptosis without affecting mitochondrial function.

**Conclusion:**

This is one of the first reports that reveal Doxorubicin and Cyclophosphamide induce significant dopaminergic neurotoxicity. Thus, Chemotherapy‐induced adverse drug reaction issues substantially persist during and after treatment and sometimes never be completely resolved clinically. Consequently, failure to adopt adequate patient care measures for cancer patients treated with certain chemotherapeutics might substantially raise the incidence of numerous movement disorders.

## 1 INTRODUCTION

The study of toxicity embraces an array of scientific areas, including anatomy, biochemistry, genetics, neuroscience, physiology, psychology, and other fields. Specifically, neurotoxicity can be an unpremeditated or accidental harmful consequence that affects the neurons and glia, leading to decreased function, degeneration, and cell death in the central and peripheral nervous system.^1^ Even though the central and peripheral nervous system has compensatory and adaptive mechanisms, exposure to exogenous xenobiotics and even endogenous ligands can result in significant neurotoxicity.^2^ Furthermore, neurons are especially vulnerable to xenobiotics‐mediated neurotoxicity because these substances can affect the permeability of the blood–brain barrier and intervene with vital neuronal functions like selective migration, neurogenesis, and apoptosis. Additionally, the risk for neurotoxicity is directly associated with the route of administration (oral or parenteral), frequency and duration (acute or chronic) of exposure, as well as metabolism (less or extensive), and the ability to pass through the blood–brain barrier.^3^ Finally, compared to the toxicity of other cells, tissues, and organ systems in the body, the neurons of the central and peripheral nervous systems are more sensitive to various insults and injuries because of their inadequate ability to regenerate effectively. According to the World Health Organization (WHO), drug‐induced Iatrogenesis is any noxious, unintended, and undesired effect of a diagnostic, preventive, or therapeutic medicine that occurs at doses used in humans for prophylaxis, diagnosis, and treatment.^4^ Neurotoxicity and neurodegeneration are important adverse health effects of numerous endogenous ligands, environmental contaminants and occupational chemicals, natural toxins, nutraceuticals, dietary supplements, cosmeceuticals, and pharmaceuticals.

Novel chemotherapeutic drugs and more effective treatment protocols have increased patient survival and decreased the chance of cancer recurrence. Cancer treatment differs depending on its origin and can be accomplished by many treatments, such as chemotherapy, radiation, surgical removal, immunotherapy, hormone therapy, or stem cell/bone marrow transplant. Alkylating drugs, nitrosoureas, antimetabolites, antitumor antibiotics, plant alkaloids, biological response modifiers, and hormonal anticancer agents are the principal chemotherapeutic classes available.^5^, ^6^ Cancer patients are frequently exposed to polypharmacy, which usually leads to Iatrogenesis‐toxicity induced as a result of medical treatment. In contrast, the higher survival rate after chemotherapy is not without serious financial effects and Iatrogenesis.^7^ It is also impossible to overstate how negatively Iatrogenesis affects the quality of life.^4^

Human physiology exhibits a number of disorders that are correlated with one another, with the development of one illness causing the other to worsen.^8^ Chemobrain is a term used to describe a persistent cognitive impairment that occurs during or after chemotherapy. Patients may have trouble maintaining their attention, memory, or processing speed. There is mounting evidence that chemobrain affects many breast cancer patients, either as a direct result of the illness or for unknown reasons; over 75% of these patients show dyscognition shortly after beginning chemotherapy, for example Referrence 9. On the other hand, a number of clinical studies demonstrate that within a few months of starting chemotherapy, cancer patients start to experience symptoms related to movement disorder.^10^, ^11^, ^12^

Chemotherapy is widely used clinically not only for curing but also for prolonging life or relieving symptoms, known as palliative chemotherapy. Various indications or clinical applications of chemotherapeutics include breast cancer, Hodgkin's lymphoma, non‐Hodgkin's lymphoma, germ cell tumor, stomach cancer, bladder cancer, lung cancer, colorectal cancer, pancreatic cancer, and bone cancer. Chemotherapeutics are employed primarily for the prevention of cell division and further cancer cell or tumor growth and do so by interfering with cell proliferation or inducing apoptosis.^13^ According to their different mechanisms of action, chemotherapeutics are classified as cell‐cycle specific drugs and cell‐cycle nonspecific drugs. Currently, chemotherapeutics used clinically include alkylating agents; plant alkaloids, antimetabolites; topoisomerase inhibitors; antitumor antibiotics (Doxorubicin, actinomycin, and Bleomycin), tyrosine kinase inhibitors, monoclonal antibodies and others.^14^, ^15^ At therapeutic doses, chemotherapeutics cause avoid adverse drug reactions, which are harmful and unintended. A literature review indicates that chemotherapeutic drugs can cause various adverse effects, including cardio‐vessel damage, hepatic toxicity, mucosal injury, inflammation, apoptosis, myotoxicity, dermal toxicity, nephrotoxicity, and cognitive impairment.^16^, ^17^ Our previous studies also confirmed the neurotoxicity associated with using the chemotherapeutic Doxorubicin.^18^, ^19^ However, there are very few reports on the direct effect of Doxorubicin and Cyclophosphamide on dopaminergic neurons currently. Thus, the current research focused on assessing the effect of Doxorubicin and cyclophosphamide on dopaminergic neurons.^20^

## 2 MATERIALS AND METHODS

### 2.1 Chemical and Reagents

Penicillin–Streptomycin and Trypsin–EDTA solutions, Fetal Bovine Serum, Dulbecco's Modified Eagle Medium (DMEM) were acquired from ATCC. Tokyo Chemical Industrial USA supplied the 3‐(4, 5‐dimethylthiazol‐2‐ 31 yl)‐2, 5‐diphenyltetrazolium bromide (MTT) reagent. Nicotinamide Adenine Dinucleotide Hydrogen (NADH), Dimethylsulfoxide (DMSO), Pyrogallol, Hydrogen Peroxide (H_2_O_2_), Phosphoric Acid, o‐phthalaldehyde (OPT), L‐Glutathione Reduced, Phenylmethanesulfonyl Fluoride (PMSF), and 2′, 7‐dichlorofluorescindiacetate (DCF‐DA) were acquired from Sigma Aldrich (St. Louis, MO). Protein quantitation was performed using Thermo Scientific Pierce 660 nm, Pierce, Rockford, IL.

### 2.2 Rat dopaminergic neurons

The rat dopaminergic neurons (N27) neurons were cultured in DMEM media with 10% fetal bovine serum and 1% penicillin–streptomycin solution. N27 neurons were generously given by Dr. Bobby Thomas, Professor, Medical University of South Carolina, Charleston, South Carolina. These neurons were grown in 75 cm^2^ flasks at 37°C and 5% CO_2_ for the MTT experiment. Trypsinization was used to separate the neurons when they had achieved 80% confluency, and they were then planted at a density of 1 × 10^5^ neurons per well.^21^

### 2.3 Treatment design

N27 neurons were treated with various concentrations of Doxorubicin (Dox: 0.5, 1, 10, 100, 500, 1000 nM) and Cyclophosphamide (Cypx: 0.02, 0.2, 2, 20, 200, 2000 μM) for 24 and 42 h. DMSO was used as a vehicle and used as a control. Based on the neuronal viability study, the doses chosen in the current study represent a nontoxic dose (low dose, had no effect on the dopaminergic neuronal viability) and a toxic dose (high dose, a dose that significantly decreases the dopaminergic neuronal viability), 500pM (as a Low Dose‐LD) and 500 nM (as a High Dose‐HD) of Doxorubicin doses and 2 mM of cyclophosphamide dose were treated for 24 h. Freshly made drug solutions were used for every experiment. Control neurons were exposed to media alone.

### 2.4 Cytotoxicity assay

Cytotoxicity of the drugs was evaluated using the MTT neuronal viability test.^21^ An Axiovert 25 inverted microscope fitted with a Nikon Coolpix 4500 camera was used to take images of the Neurons.

### 2.5 Protein assay

Using a Protein Assay reagent kit (Thermo Scientific Pierce 660 nm, Pierce, Rockford, IL), the colorimetric procedure was used to assess the protein, the process, by which a particular dye‐metal complex binds to protein in an acidic environment alters the dye's maximum absorption wavelength (660 nm). Bovine serum albumin^22^ was utilized for standardization.^23^, ^24^

### 2.6 Mitochondrial Complex‐I activity

The mitochondrial Complex‐I activity was performed spectrophotometrically using NADH as the substrate. Complex‐I (NADH dehydrogenase) in the mitochondria catalyzes the conversion of NADH oxidation to NAD+. A standard curve was obtained using NADH.^23^, ^25^, ^26^, ^27^

### 2.7 Mitochondrial Complex‐IV activity

The mitochondrial Complex‐IV activity was performed colorimetrically using Cytochrome‐C as the substrate. Complex‐IV (Cytochrome‐C oxidase) in the mitochondria initiates the oxidation of Cytochrome‐C. A standard curve was obtained using Cytochrome‐C.^23^, ^26^

### 2.8 Quantifying reactive oxygen species

ROS content was determined by detecting the conversion of nonfluorescent chloromethyl‐DCF‐DA (2′, 7‐dichlorofluorescindiacetate, DCF‐DA) to fluorescent DCF using an excitation wavelength of 492 nm and an emission wavelength of 527 nm. This was done using spectrofluorometry.^23^, ^25^, ^26^, ^27^, ^28^

### 2.9 Lipid peroxidation

The colorimetric method using thiobarbituric acid was used to quantify the lipid peroxide content. The formed thiobarbituric acid reactive substances (TBARS) were measured using a plate reader at 532 nm.^23^, ^26^, ^28^

### 2.10 Nitrite assay

The nitrite content was measured using the Griess reagent, where nitrite reacts with sulfanilamide under acidic conditions to form a diazonium ion that can be measured spectrophotometrically at 545 nm.^23^, ^25^, ^27^, ^28^

### 2.11 Glutathione content

Glutathione reacts with non‐fluorescent‐OPT and forms a fluorescent substance, which was measured spectrofluorometrically (340 nm/420 nm).^23^, ^25^, ^26^, ^28^

### 2.12 Activity of glutathione peroxidase

The reduction of NADPH was measured spectrophotometrically (340 nm) to assess glutathione peroxidase (GSH‐Px) activity. A standard curve was obtained using NADPH.^28^

### 2.13 Activity of Catalase

Catalase activity was measured using hydrogen peroxide as the substrate. Activity determined by spectrophotometrically tracking the breakdown of hydrogen peroxide at 240 nm. A standard curve was obtained using hydrogen peroxide.^25^, ^29^

### 2.14 Activity of superoxide dismutase

SOD activity was measured using pyrogallol as the substrate. The activity of superoxide dismutase was measured at 420 nm and reported as a percentage change from the control.^29^

### 2.15 Quantitative RT‐PCR and BAX expression

Reverse transcription was performed with the QuantiTect Reverse Transcription Kit (Qiagen), and quantitative PCR was performed by using the QuantiTect SYBR Green Kit (Qiagen) and iCycler iQ Real‐Time PCR Detection System (Bio‐Rad; Hercules, CA) according to the manufacturer's protocol and gene‐specific primers of 18S rRNA (housekeeping gene) primers (forward: 5′‐ GGTTGTCGCCCTTTTCTA‐3′ and reverse: 5′‐ CGGAGGAAGTCCAATGTC‐3′). Reverse transcription (RT) was performed for 30 min at 50°C and 15 min at 95°C. The specific gene expression was normalized to the level of 18S rRNA.^30^

### 2.16 Statistical analysis

Results were expressed as average ± standard error mean (SEM). One‐way analysis of variance (ANOVA) followed by Dunnet's multiple comparisons tests (*p < .05 was considered to be statistically significant) was used in the current study (Prism‐V software; La Jolla, CA, USA).^23^, ^29^

## 3 RESULTS

### 3.1 Dopaminergic neurotoxicity mediated by chemotherapeutics (Doxorubicin and Cyclophosphamide)

**FIGURE 1:**
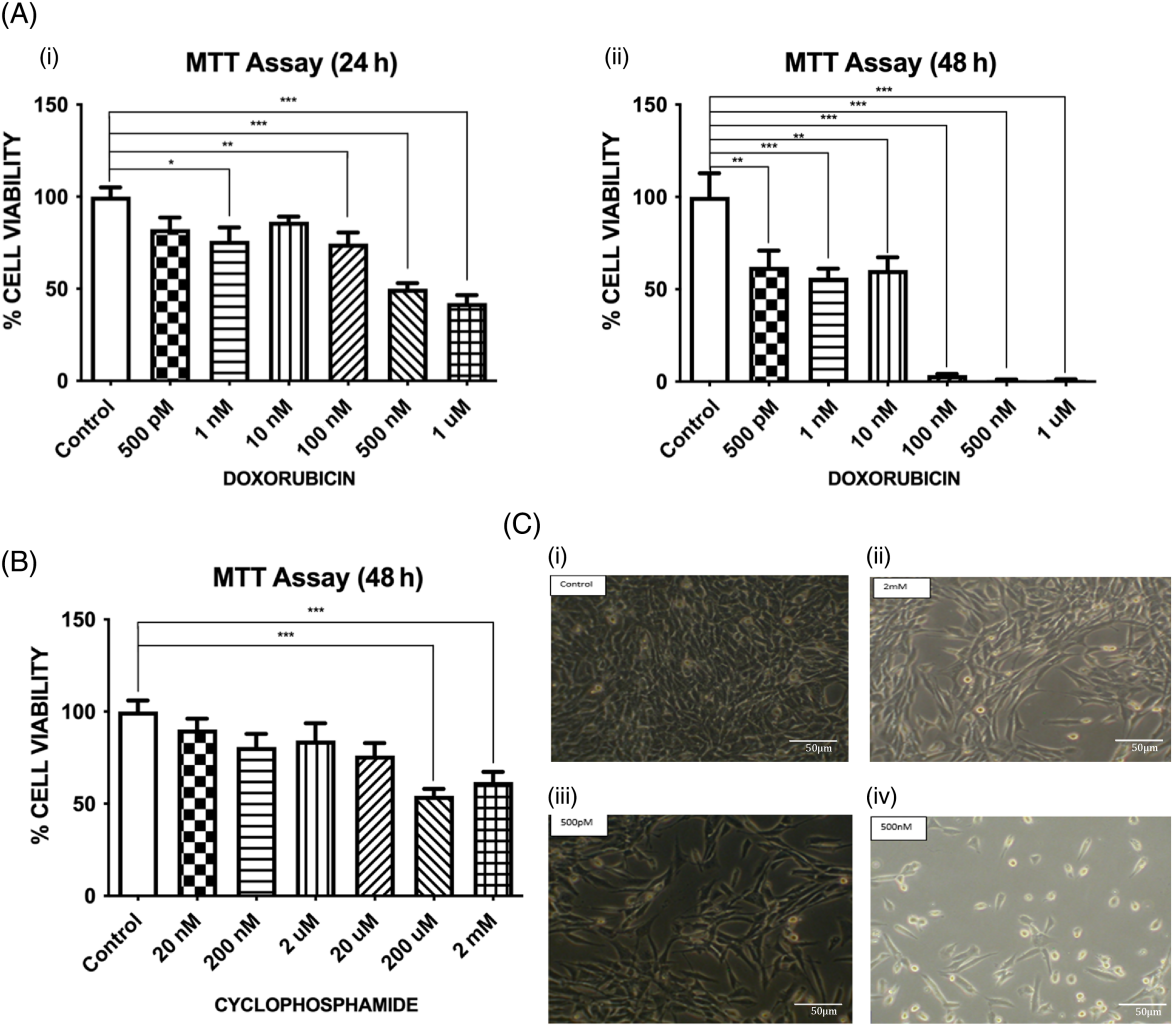
(A) (i) Effect of Doxorubicin 24 h incubation on N27 rat dopaminergic neuronal viability: N27 dopaminergic neurons were treated with different doses of Doxorubicin and incubated for 24 h at 37°C. Cell viability was evaluated using the MTT reduction assay (*n* = 12). Results are expressed as percentage control ± SEM. Doxorubicin (1 nM, 100 nM, 200 nM, and 1 μM) significantly decreased the dopaminergic neuronal viability as compared to the control (*n* = 12, *p* < .05). Statistical comparisons were made using one‐way ANOVA/Dunnett's multiple comparison test. Note (*) indicates a statistically significant difference when compared to controls. (A) (ii): Effect of Doxorubicin 48 h incubation on N27 rat dopaminergic neuronal viability: N27 dopaminergic neurons were treated with different doses of Doxorubicin and incubated for 48 h at 37°C. Cell viability was evaluated using the MTT reduction assay (*n* = 12). Results are expressed as percentage control ± SEM. Doxorubicin (500pM, 1 nM, 100 nM, 500 nM, and 1 μM) significantly decreased the dopaminergic neuronal viability as compared to the control (*n* = 12, *p* < .05). Statistical comparisons were made using one‐way ANOVA/Dunnett's multiple comparison test. Note (*) indicates a statistically significant difference when compared to controls. (B) Effect of Cyclophosphamide 24 h incubation on N27 rat dopaminergic neuronal viability: N27 dopaminergic neurons were treated with different doses of Cyclophosphamide and incubated for 48 h at 37°C. Cell viability was evaluated using the MTT reduction assay (*n* = 12). Results are expressed as percentage control ± SEM. Cyclophosphamide (200 and 2 mM) significantly decreased the dopaminergic neuronal viability as compared to the control (*n* = 12, *p* < .05). Statistical comparisons were made using one‐way ANOVA/Dunnett's multiple comparison test. Note (*) indicates a statistically significant difference when compared to controls. (C) Effect of Doxorubicin and Cyclophosphamide on N27 neurons morphological changes: Morphological changes in N27 neurons following the treatment with Doxorubicin [500pM ((E) iii) and 500 nM (E).iv)] and Cyclophosphamide [2 mM ((E).ii)] at 24 h. Cypx, Cyclophosphamide; LD dox, Low Dose of Doxorubicin; HD dox, High Dose of Doxorubicin.

N27 neurons were exposed to different doses of Doxorubicin (0.5, 1, 10, 100, 500, 1000 nM) and cyclophosphamide (0.02, 0.2, 2, 20, 200, 2000 μM) for 24 and 48 h time point. Both the chemotherapeutics induced significant dose and time‐dependent decrease in neuronal proliferation as compared to the control (*n* = 12, *p < .05, Figure 1A(i,ii),B). However, Cyclophosphamide exhibited significantly lower neurotoxicity as compared to Doxorubicin. Cyclophosphamide alone did not have any significant effect on dopaminergic neuronal proliferation at 24 h (data not shown), but there was a significant dose‐dependent decrease in dopaminergic neuronal proliferation at 48 h (Figure 1b). Doxorubicin and Cyclophosphamide induced neuroanatomical deficits, such as structural alterations and deformation as seen by dopaminergic neurons morphologically shrinking, followed by rounding of the neurons (loss of the structure), and ultimately leading to drastic synaptic destructions (Figure 1C(i–iv)).

### 3.2 Doxorubicin and cyclophosphamide did not affect Mitochondrial Function (Complex‐I activity and Complex‐IV activities)

**FIGURE 2:**
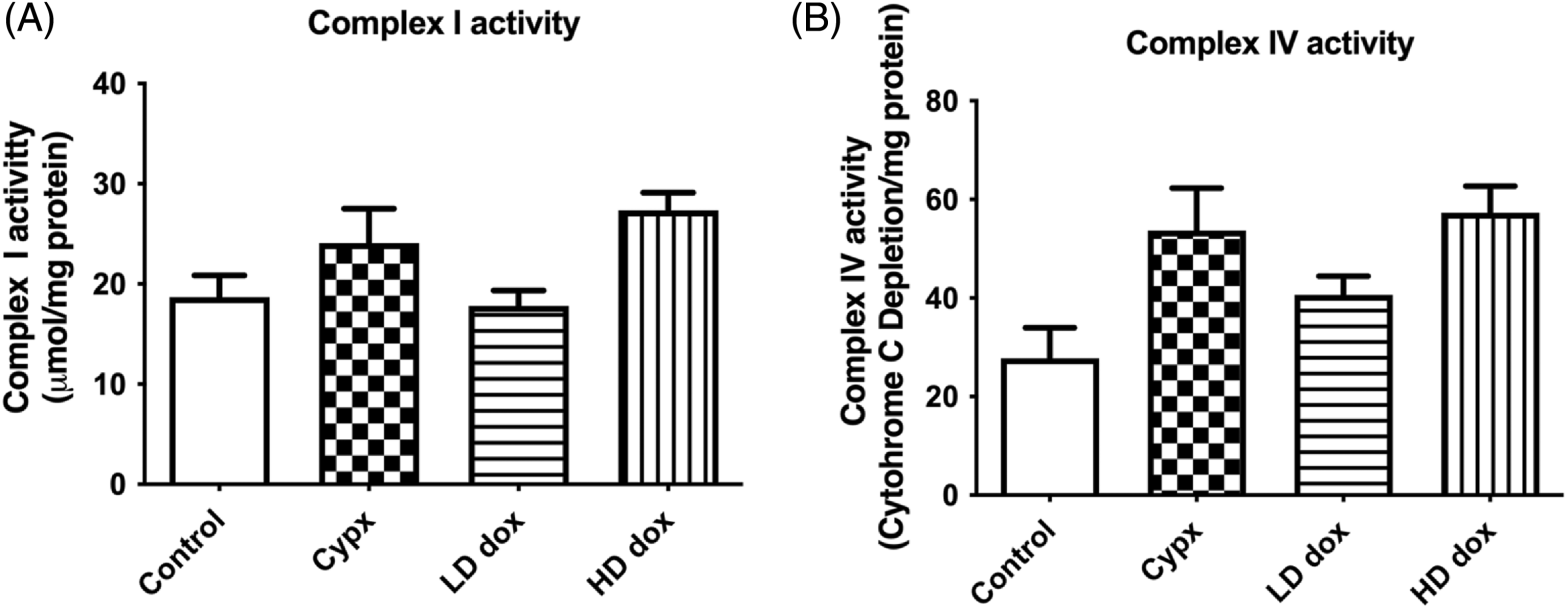
(A) Effect of Cyclophosphamide and Doxorubicin on Mitochondrial Complex‐I activity in N27 cells. Complex‐I activity was measured spectrophotometrically (340 nm) using NADH as substrate. Doxorubicin and Cyclophosphamide did not have any significant effect on Complex‐I activity. Results are expressed as NADH oxidized (μM)/mg protein, mean ± SEM. Statistical comparisons were made using one‐way ANOVA/Dunnet's multiple comparison test. Note (*) indicates a statistically significant difference when compared to controls. (B) Effect of Cyclophosphamide and Doxorubicin on Mitochondrial Complex‐IV activity in N27 cells. Complex‐IV activity was measured colorimetrically using cytochrome‐C as substrate. Doxorubicin and Cyclophosphamide did not have any significant effect on the mitochondrial Complex‐IV activity (*n* = 5). Results are expressed as cytochrome c oxidized (μM)/mg protein, mean ± SEM. Statistical comparisons were made using one‐way ANOVA/Dunnet's multiple comparison test. Note (*) indicates a statistically significant difference when compared to controls. Cypx, cyclophosphamide; LD dox, low dose of doxorubicin; HD dox, high dose of doxorubicin; GSH, glutathione; SOD, superoxide dismutase.

Chemotherapeutics, doxorubicin, and cyclophosphamide at the present dose and time point did not affect the mitochondrial Complex‐I (Figure 2A) and Complex‐IV activities (Figure 2B).

### 3.3 Doxorubicin and cyclophosphamide increase BAX expression

**FIGURE 3:**
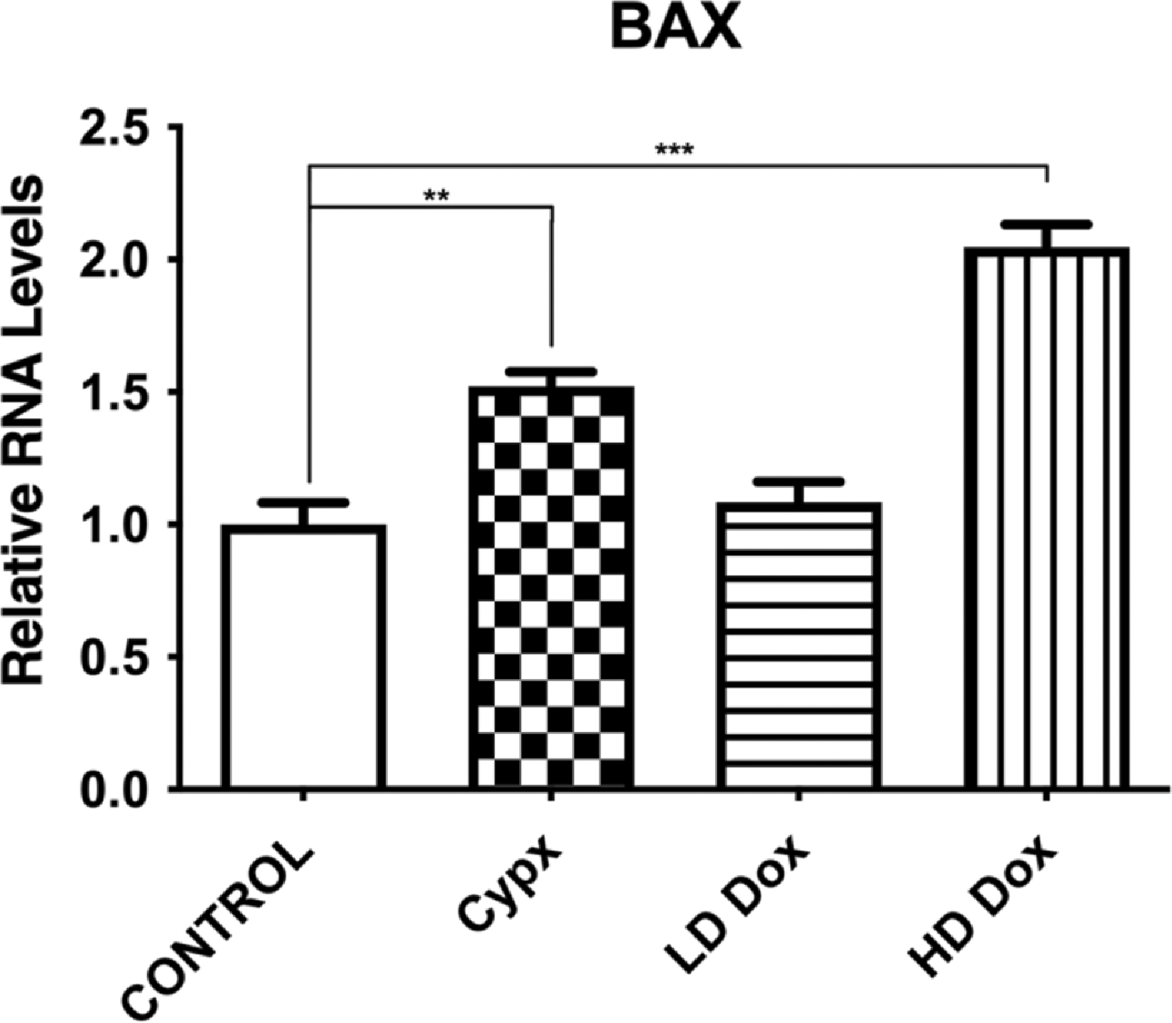
Doxorubicin (500 nM‐HD) and Cyclophosphamide (2 mM) showed a significant increase in BAX expression in N27 cells compared to the control (**p < .01, ***p < .001, *n* = 3). Significance was measured using GAPDH control. Statistical comparisons were made using one‐way ANOVA/Dunnet's multiple comparison test. Note (*) indicates a statistically significant difference when compared to controls. Cypx, cyclophosphamide; LD dox, low dose of doxorubicin; HD dox, high dose of doxorubicin; ROS, reactive oxygen species; RFU, relative fluorescence unit.

Regarding the apoptotic marker BAX, RT‐PCR showed a significant increase by Cyclophosphamide by 52% and Doxorubicin (500 nM) by 104% as compared to control (*n* = 3, *p < .0001, Figure 3).

### 3.4 Doxorubicin and cyclophosphamide significantly affect prooxidants (ROS, Nitrite, and Lipid peroxide)

#### 3.4.1 Doxorubicin and cyclophosphamide increase ROS generation

**FIGURE 4:**
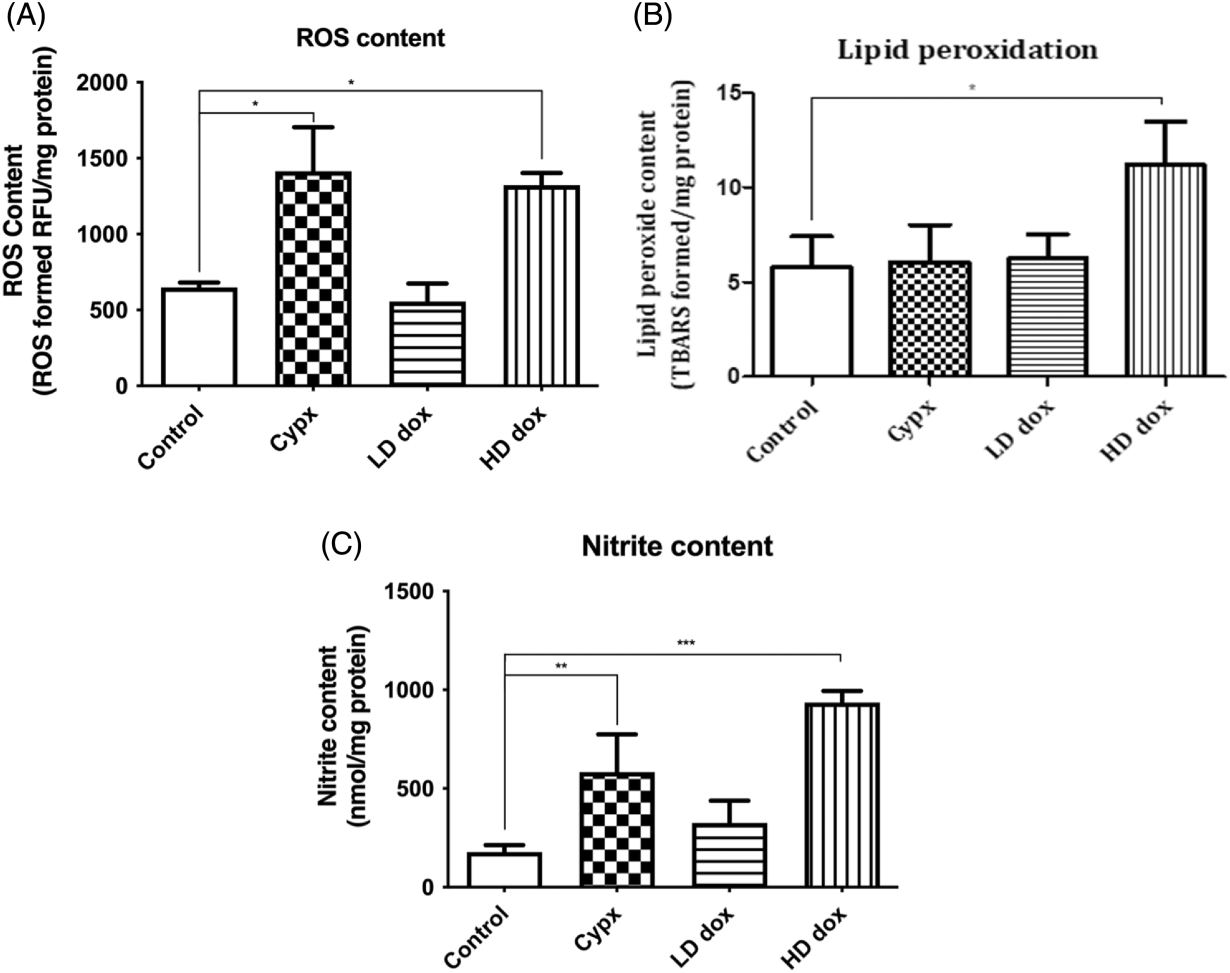
(A) Effect of chemotherapeutics on ROS generation in N27 cells. Cyclophosphamide and Doxorubicin cause oxidative stress by generating reactive oxygen species in N27 cells after 24 h. The fluorescent product DCF was measured spectrofluorometrically. ROS was measured as relative fluorescence units (492/527 nm)/mg protein. Cyclophosphamide (2 mM) and Doxorubicin (500 nM) showed a significant increase in ROS generation (*p < .05, *n* = 5). Results are expressed as ROS generation control ± SEM. Statistical comparisons were made using one‐way ANOVA/Dunnet's multiple comparison test. Note (*) indicates a statistically significant difference when compared to controls. (B) Effect of Cyclophosphamide and Doxorubicin on N27 cells for lipid peroxide formation. Lipid peroxide was measured spectrophotometrically. Due to the increased ROS generation, Doxorubicin (500 nM) induced a significant formation of lipid peroxide (*p < .05, *n* = 5). Lipid peroxide formation was measured as TBARS formed (532 nm)/mg protein, and the result is expressed as lipid peroxide formed as compared to the control. Statistical comparisons were made using one‐way ANOVA/Dunnet's multiple comparison test. Note (*) indicates a statistically significant difference when compared to controls. (C) Effect of Cyclophosphamide and Doxorubicin on Nitrite production in N27 cells. Nitrite production was determined spectrophotometrically (540 nm) as formed Nitrite nM/mg protein. Results are expressed as Nitrite formed control ± SEM. Cyclophosphamide (2 mM) and Doxorubicin (500 nM) showed a significant increase in Nitrite production (**p < .01, ***p < .001, *n* = 5) in N27 cells after 24 h. Statistical comparisons were made using one‐way ANOVA/Dunnet's multiple comparison test. Note (*) indicates a statistically significant difference when compared to controls.

Chemotherapeutics, Doxorubicin (500 nM; dose‐dependently) significantly increased ROS generation by 103%, and Cyclophosphamide (2 mM) significantly enhanced the generation of ROS by 117% as compared to control (*n* = 5, *p = .0023 (*p* < .05); Figure 4).

#### 3.4.2 Doxorubicin and cyclophosphamide increase nitrite content

Chemotherapeutics, doxorubicin (500 nM; dose‐dependently) significantly increased nitrite content by 427%, and Cyclophosphamide (2 mM) significantly increased the nitrite formation by 228% as compared to control (*n* = 5, *p* < .05; Figure 4B).

#### 3.4.3 Doxorubicin increases lipid peroxide formation

Only the high dose of Doxorubicin (500 nM) significantly increased lipid peroxidation by 93% (*n* = 5, *p* = .01(*p* < .05), Figure 4C) as compared to control. Nonetheless, cyclophosphamide did not significantly increase the formation of lipid peroxide.

### 3.5 Effect of doxorubicin and cyclophosphamide on antioxidant markers

#### 3.5.1 Doxorubicin significantly depleted glutathione content

**FIGURE 5:**
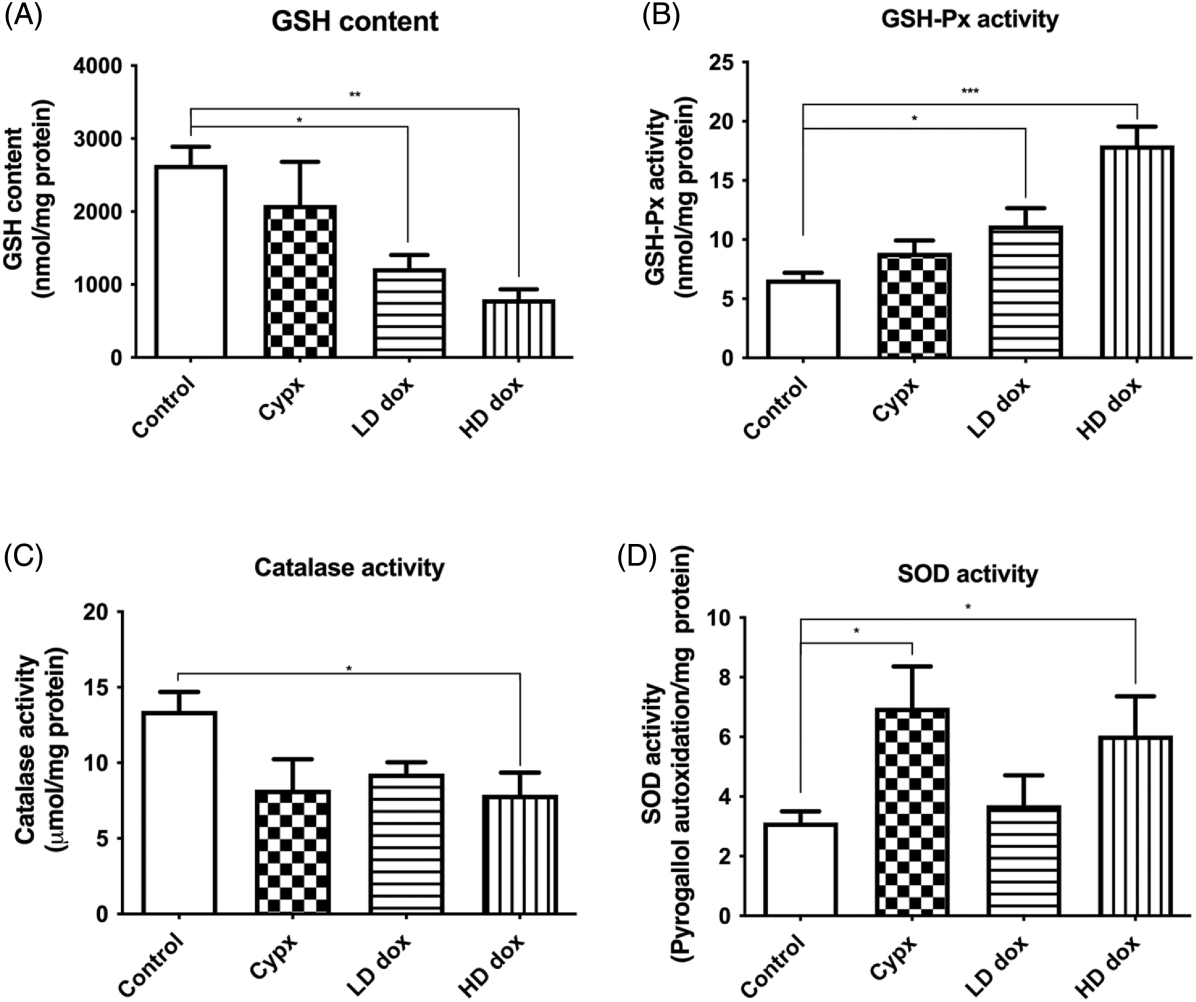
(A) Effect of Cyclophosphamide and Doxorubicin on N27 cells on glutathione content: Glutathione content was measured by the product that reacted with o‐phthalaldehyde (OPT) spectrofluorimetrically (340/420 nm). Results are expressed as GSH content (μM)/mg protein, mean ± SEM. The low dose and high dose of Doxorubicin showed significant depletion in GSH content (*p < .05, **p < .01, *n* = 5). Statistical comparisons were made using one‐way ANOVA/Dunnet's multiple comparison test. Note (*) indicates a statistically significant difference when compared to controls. (B) Effect of Cyclophosphamide and Doxorubicin on glutathione peroxidase activity in N27 cells. Glutathione peroxidase activity was measured spectrophotometrically using NADPH as substrate. Doxorubicin (500 nm) significantly increased the glutathione peroxidase activity as compared to the control (*p < .05, ***p < .001, *n* = 5). Results are expressed as NADPH oxidized nM/mg total protein, mean ± SEM. Statistical comparisons were made using one‐way ANOVA/Dunnet's multiple comparison test. Note (*) indicates a statistically significant difference when compared to controls. (C) Effect of Cyclophosphamide and Doxorubicin on Catalase activity in N27 cells. Catalase activity was measured by the depletion of Hydrogen Peroxide spectrophotometrically (240 nm). Results are expressed as hydrogen peroxide oxidized uM/mg protein, Mean ± SEM. Doxorubicin (both doses) significantly decreased the catalase activity (*p < .05, *n* = 5). Statistical comparisons were made using one‐way ANOVA/Dunnet's multiple comparison test. Note (*) indicates a statistically significant difference when compared to controls. (D) Effect of Cyclophosphamide and Doxorubicin on SOD activity in N27 cells. SOD activity was measured spectrophotometrically using pyrogallol as a substrate. The high dose (500 nm) of Doxorubicin and Cyclophosphamide increased SOD activity significantly (*p < .05, *n* = 5). Results are expressed as inhibition of pyrogallol autoxidation/mg total protein, mean ± SEM. Statistical comparisons were made using one‐way ANOVA/Dunnet's multiple comparison test. Note (*) indicates a statistically significant difference when compared to controls.

Doxorubicin (500pM by 54% and 500 nM by 70%) dose‐dependently significantly depleted the glutathione (*n* = 5, *p < .05, Figure 5A) as compared to control. Cyclophosphamide did not affect the glutathione content.

### 3.6 Augmentation of GSH‐Px activity by Doxorubicin

Doxorubicin (500pM by 68% and 500 nM by 170%) dose‐dependently significantly increased the GSH‐Px activity (*p < .0001, *n* = 5, Figure 5B). Cyclophosphamide did not affect the GSH‐Px activity.

### 3.7 Doxorubicin inhibited catalase activity

Only the high dose of doxorubicin significantly inhibited the catalase activity by 42% (*p < .05, *n* = 5, Figure 5c) as compared to control. Cyclophosphamide did not affect the catalase activity.

### 3.8 Doxorubicin and cyclophosphamide increased SOD activity

The chemotherapeutics, doxorubicin (500 nM) by 93% and cyclophosphamide by 123%, significantly increased the SOD activity (*n* = 5, *p < .05, Figure 5D).

## 4 DISCUSSION

Most cancer treatments are associated with adverse reactions, several of which are severe. The risks and improvements to treatment, including those of iatrogenic illnesses from therapy, are tolerable with careful therapy selection. Chemotherapy‐related neurotoxicity is becoming more often identified, with 60% of patients receiving chemotherapy drugs with neurotoxic potential.^31^ Furthermore, the risk of neurotoxicity is expected to increase when two or more neurotoxic drugs are taken in combination. High‐dose treatment, diabetes mellitus, alcoholism, and prior or concurrent use of other neurotoxic medications are all risk factors that increase a patient's chance of developing chemotherapeutic‐related neurotoxicity.^31^, ^32^, ^33^ Cognitive impairments, seizures, insomnia, tremors, ataxia, other movement & mental abnormalities, or even comas (albeit rare) are the major CNS toxicities that are frequently noticed.^34^, ^35^ Endogenous and exogenous neurotoxins cause dopaminergic neuronal insults by causing oxidative stress (ROS and quinones formation, antioxidant exhaustion, and upsurge of proxidant content and activities), decreased function of mitochondria (Complex‐I and Complex‐IV inhibition, decreased formation of ATP), and programmed cell death/apoptosis (rise in pro‐apoptotic and decline in anti‐apoptotic ligands). The cellular organelle, mitochondria, are considered a potent controller of cell/neuronal differentiation and growth due to the formation of ATP. Aging and other neurodegenerative health ailments (Alzheimer's, Huntington's, and Parkinson's disease) generally occur due to decreased mitochondrial functions, which are associated with the specific deficiency of mitochondrial Complex‐I and/or IV activities.^36^ Regarding oxidative stress, lipid peroxide formation is a chain cellular consequential mechanism, in which ROS attacks the polyunsaturated fatty acids, leading to oxidative degradation of lipids.^37^, ^38^ Enhanced production of ROS and decreased ROS scavenging can lead to aging and numerous other diseases (Atherosclerosis, Cancer, Alzheimer's, Huntington's, and Parkinson's disease). ROS can directly damage nucleic acids (DNA/RNA), proteins (amino acids), and lipids that are essential for neuronal‐glial activities.^39^

In general, the exogenous and endogenous neurotoxins readily cross the blood–brain barrier and cause bilateral lesions of the neurons, resulting in the depletion of monoamines (dopamine, norepinephrine, and serotonin) in the specific brain regions. Lesions of insults in the dopaminergic neurons of the nigrostriatal tract can lead to Parkinsonian‐like syndrome (akinesis, bradykinesia, rigidity, postural instability, tremor, and freezing). Dopaminergic neurotoxins mostly bind with enzymes associated with dopamine synthesis and metabolism (tyrosine hydroxylase, aromatic amino acid decarboxylase, catechol‐O‐methyl transferase, and monoamine oxidase). Hydrogen peroxide, divalent metals, 6‐hydroxydopamine (6‐OHDA), 1–95 methyl‐4‐phenyl‐1,2,3,6‐tetrahydropyridine (MPTP), rotenone, and herbicides (paraquat/diquat) are the most prevalent endogenous and exogenous dopaminergic neurotoxins.^40^ A few of these neurotoxins share structural similarities with monoamines such as norepinephrine and dopamine. Numerous catecholaminergic plasma membrane transporters, including dopamine (DAT) and norepinephrine (NET) transporters, are strongly targeted by these neurotoxins.^41^ Furthermore, dopaminergic neurotoxins can penetrate the membranes of organelles, resulting in accumulating inside a specific organelle. In the powerhouse of the cell (mitochondria), dopaminergic neurotoxins specifically inhibit the mitochondrial transport chain, synthesis of microtubules from tubulin, and changes in ion channels on dorsal root ganglia and dorsal horn, resulting in neuronal death.^42^

Chemotherapeutics‐related cognitive impairment (CRCI) has become an area of great research interest in the past few years. Chemotherapeutics can impair cognitive performance by decreasing neurogenesis and inducing excitotoxicity, inflammation, oxidative stress, mitochondrial dysfunction, and apoptosis in the hippocampus and cortex.^32^, ^43^ Currently, effective interventions for patients with cancer with CRCI are also lacking, as are guidelines on how to care for older patients with cancer and CRCI. Chemotherapeutics induce significant adverse drug reactions and hypersensitivity reactions. Alarmingly, in the past decade, there have been increased reports of chemotherapeutics‐induced neurotoxicity around the world. Cisplatin, carmustine, and ifosfamide readily cross the blood–brain barrier. However, Doxorubicin does not achieve significant concentrations in the CNS due to efflux by p‐glycoprotein. However, when Doxorubicin is used with Ondansetron (block the p‐glycoprotein efflux pump) regularly to suppress nausea/vomiting can significantly increase its CNS levels.^44^

Based on our previous studies and the existing literature, this study may be one of the initial research studies related to the effect of Doxorubicin and Cyclophosphamide on dopaminergic neurons. In the current study, the current dose and duration of exposure of chemotherapeutics (Doxorubicin and Cyclophosphamide) decreased antioxidants, increased proxidants, and increased BAX (pro‐apoptotic) expression in the dopaminergic neurons without affecting the mitochondrial functions. Oxidative stress occurred due to the significant increase in the formation of ROS and nitrite that caused lipid peroxidation. When dopamine is overmetabolized and exposed to neurotoxins, hydrogen peroxide is produced, which subsequently results in the production of reactive oxygen species (ROS), which in turn causes oxidative stress and the degeneration of healthy cells, is the primary cause of health conditions such as aging, atherosclerosis, and the majority of neurological illnesses. Numerous biological components, including proteins, lipids, and nucleic acids (DNS/RNA), are known to be damaged by ROS, which is necessary for regular physiological processes.^39^ Few studies demonstrated that, an upsurge in ROS species could trigger the ERK pathway that damages neural cells.^43^, ^45^ Doxorubicin and Cyclophosphamide significantly reduced the content of the potent antioxidant glutathione and decreased catalase activity. It has been demonstrated that cyclophosphamide, an alkylating chemotherapy agent, influences the factor linked with the tumor necrosis factor α (TNF‐α) receptor.^46^, ^47^ This results in reduced expression of the anti‐apoptotic Bcl‐2 protein and increased expression of the pro‐apoptotic BAX protein. The following subsequently activates caspases, which cause neurodegeneration.^48^, ^49^ Normally, dopaminergic neurodegeneration and neurotoxicity are associated with aging, genetics, and exposure to various toxins, but this study emphasizes the role of prevalent clinically approved drugs‐induced dopaminergic neurotoxicity. In spite of its therapeutic value, clinically approved drugs cause reversible or irreversible toxicity by affecting the morphology and/or function of the cells. Globally, due to their pharmacodynamic and pharmacokinetic properties, anthracycline and alkylating cytophosphane chemotherapeutics are still indicated for serval types of cancer. However, the toxicity profile on the dopaminergic neurotransmission of these drugs are not well clinically monitored. Based on our findings, anthracycline and alkylating cytophosphane drugs can induce dopaminergic neuronal insults, which can increase the risks for various dopaminergic ailments (movement, mental, memory, and endocrine disorders).

## 5 CONCLUSION

**FIGURE 6:**
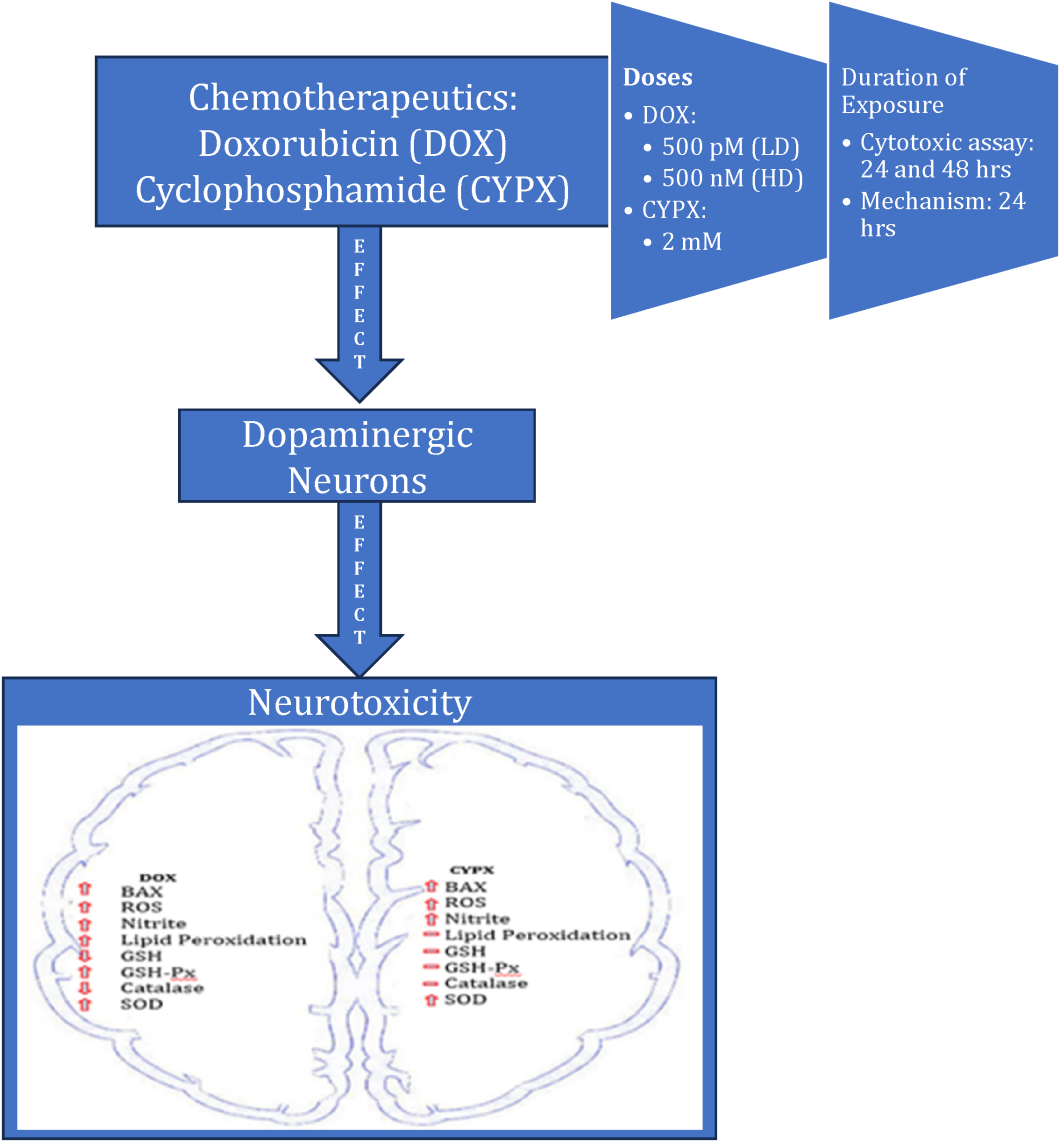
In vitro effects of Doxorubicin and Cyclophosphamide on Dopaminergic Neurons. Cypx, cyclophosphamide; LD dox, low dose of doxorubicin; HD dox, high dose of doxorubicin.

Chemotherapeutics‐induced iatrogenesis occurs significantly both during and after treatment, which may never fully resolve clinically. Moreover, the chemotherapeutics‐induced CNS pathologies are depression, epilepsy, infarctions, palsy, neuropathy, leukoencephalopathy, ototoxicity and loss of memory. Thus, there is an imminent requirement to understand the etiologies associated with Chemotherapeutics‐induced iatrogenesis that can lead to novel preventive and therapeutic strategies. Therefore, our present in vitro study (Figure 6) on dopaminergic neurons suggests Doxorubicin and Cyclophosphamide may cause significant dopaminergic neurotoxicity. Consequently, failing to implement effective patient care strategies for cancer patients treated with Doxorubicin and Cyclophosphamide can significantly enhance the occurrence of various dopaminergic disorders.

## AUTHOR CONTRIBUTIONS

**Darshini Desai:** Data curation (lead); methodology (lead); software (lead); writing – original draft (lead). **Mohammed Majrashi:** Methodology (lead); software (lead); validation (lead); writing – original draft (equal). **Suhrud Pathak:** Methodology (equal); validation (equal); writing – original draft (equal); writing – review and editing (equal). **Mohammed Almaghrabi:** Data curation (equal); methodology (equal); software (equal). **Keyi Liu:** Methodology (supporting); writing – original draft (supporting). **Satyanarayana R. Pondugula:** Investigation (equal); supervision (equal); writing – review and editing (equal). **Amit K. Tiwari:** Supervision (equal); writing – review and editing (equal). **R. Jayachandra Babu:** Investigation (equal); supervision (equal); writing – review and editing (equal). **Jack Deruiter:** Supervision (equal); writing – review and editing (equal). **Muralikrishnan Dhanasekaran:** Conceptualization (equal); investigation (equal); supervision (equal); validation (equal); visualization (equal); writing – review and editing (equal).

## Data Availability

Data sharing is not applicable to this article as no new data were created or analyzed in this study.
